# A graphic elicitation technique to represent patient rights

**DOI:** 10.1186/s13031-020-00331-8

**Published:** 2020-12-14

**Authors:** Catherine R. McGowan, Nora Hellman, Louisa Baxter, Sonali Chakma, Samchun Nahar, Ahasan Ud Daula, Kelly Rowe, Josie Gilday, Patricia Kingori, Rachel Pounds, Rachael Cummings

**Affiliations:** 1 Humanitarian Public Health Technical Unit, Save the Children UK, 1 St John’s Lane, London, EC1M 4AR UK; 2 Department of Public Health, Environments & Society, London School of Hygiene & Tropical Medicine, 15-17 Tavistock Place, London, WC1H 9SH UK; 3 Save the Children International, Rohingya Response, Cox’s Bazaar, Bangladesh; 4 Save the Children Australia, 33 Lincoln Square South, Carlton, VIC 3053 Australia; 5 Save the Children International, St Vincent House, 30 Orange Street, London, WC2H 7HH UK; 6 Nuffield Department of Population Health, University of Oxford, Old Road Campus, Roosevelt Drive, Oxford, OX3 7LF UK

**Keywords:** Graphic elicitation, Patient charter, Patient rights, Rohingya, FDMN, Governance

## Abstract

**Background:**

A patient charter is an explicit declaration of the rights of patients within a particular health care setting. In early 2020 the Save the Children Emergency Health Unit deployed to Cox’s Bazar Bangladesh to support the establishment of a severe acute respiratory infection isolation and treatment centre as part of the COVID-19 response. We developed a charter of patient rights and had it translated into Bangla and Burmese; however, the charter remained inaccessible to Rohingya and members of the host community with low literacy.

**Methods:**

To both visualise and contextualise the patient charter we undertook a graphic elicitation method involving both the Rohingya and host communities. We carried out two focus group discussions during which we discussed the charter and agreed how best to illustrate the individual rights contained therein.

**Results:**

Logistical constraints and infection prevention and control procedures limited our ability to follow up with the original focus group participants and to engage in back-translation as we had planned; however, we were able to elicit rich descriptions of each right. Reflecting on our method we were able to identify several key learnings relating to: 1) our technique for eliciting feedback on the charter verbatim versus a broader discussion of concepts referenced within each right, 2) our decision to include both men and women in the same focus group, 3) our decision to ask focus group participants to describe specific features of each illustration and how this benefited the inclusivity of our illustrations, and 4) the potential of the focus groups to act as a means to introduce the charter to communities.

**Conclusions:**

Though executing our method was operationally challenging we were able to create culturally appropriate illustrations to accompany our patient charter. In contexts of limited literacy it is possible to enable access to critical clinical governance and accountability tools.

## Background

A patient charter is an explicit declaration of the rights of patients within a particular health care setting. Patient charters often consolidate existing substantive or procedural rights that are provided for according to legal statutes, ethical principles, and professional commitments. A patient charter is intended to improve both the quality and accessibility of care by increasing accountability and transparency; it is a means of encouraging good clinical governance, and an important hallmark of an earnest commitment to accountability. Patient charters signal a move towards patient-centred care and are a common feature of both public and private health care systems; some examples include the Australian Charter of Healthcare Rights, The Kenya National Patient’s Rights Charter, and the National Health Service (NHS) Constitution for England [1–3]. Despite the growing emphasis on patient charters as a critical component of both clinical governance and accountability they have been adopted by few humanitarian health actors.

Save the Children’s Emergency Health Unit (EHU) was established in 2015 with the aim of increasing the predictability, speed, and quality of Save the Children’s health response in humanitarian emergencies. In 2018 the EHU was deployed to North East Syria to provide health services for internally displaced populations fleeing military operations in and around Al-Raqqa. The Clinical Lead developed a package of accountability mechanisms for the response based on an established internal Save the Children checklist; however, as part of the after action review (AAR) the checklist was deemed to have fallen short as it did not include a patient charter. The AAR included the recommendation that the EHU develop a patient charter and display it prominently, in relevant languages, within health facilities [4].

In early 2020 the EHU was deployed to Camp 21, Cox’s Bazar, Bangladesh to establish a severe acute respiratory infection isolation and treatment centre (SARI ITC) as part of the COVID-19 response. The team reached out to other NGO health actors both on the ground and at headquarters, but none were aware of any existing patient charters or other statements of patient rights or guidelines for developing these for humanitarian contexts. The responsibility for developing the patient charter fell within both the Save the Children clinical quality framework and the monitoring, evaluation, accountability, and learning (MEAL) function. As such, the team worked collaboratively to develop a patient charter for the EHU (which would be included within an existing package of accountability tools) with the intention of displaying it in the SARI ITC. We carried out an internet search for existing charters and deemed the NHS Scotland Charter of Patient Rights and Responsibilities to best consolidate the humanitarian principle of impartiality (e.g. the right not to be discriminated against), the standard accountability provision within Save the Children (e.g. the right to have access to a feedback and response mechanism), as well as the basic rights of patients in any healthcare setting (e.g. the right to privacy and confidentiality) [5]. We developed a patient charter (Table 1) based on the four topics defined in the NHS Scotland charter: 1) accessing and using services, 2) communication and patient involvement, 3) privacy and confidentiality, and 4) feedback and complaints.

**Table 1:** Patient charter

As a patient in this healthcare centre you have the following rights: 1. The right not to be discriminated against because of age, gender, disability, political beliefs, refugee status, or ethnic background 2. The right to receive respectful and dignified care 3. The right to receive high quality care; care that is safe, effective, efficient, timely, and equitable 4. The right to receive care at no cost 5. The right to be informed about and involved in decision making on your plan of care 6. The right to privacy and confidentiality; your health information will be protected 7. The right to share concerns and have them addressed; and have access to a feedback and response mechanism

**Fig. 1:**
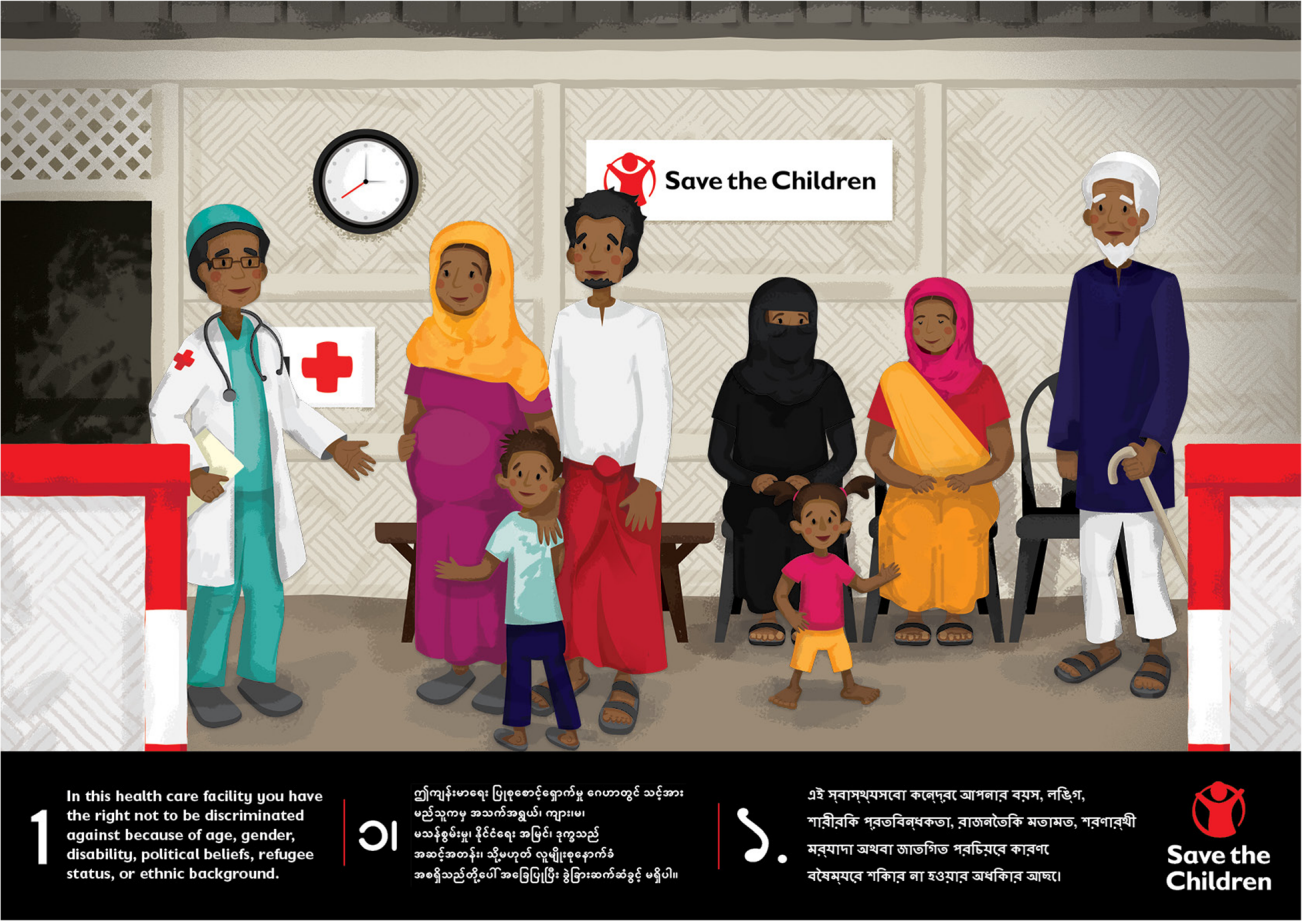
In this health care facility you have the right not to be discriminated against

**Fig. 2:**
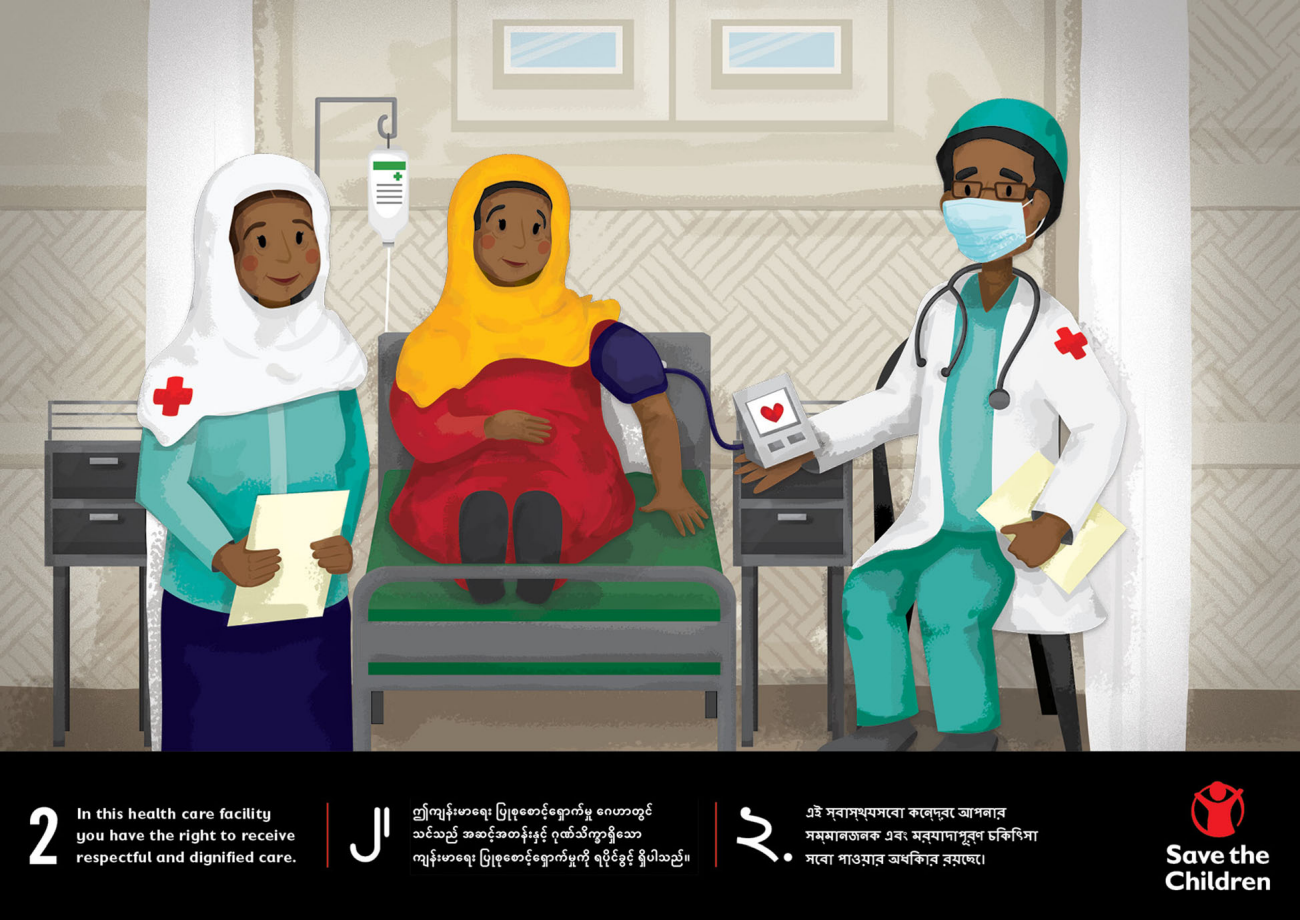
In this health care facility you have the right to receive respectful and dignified care

**Fig. 3:**
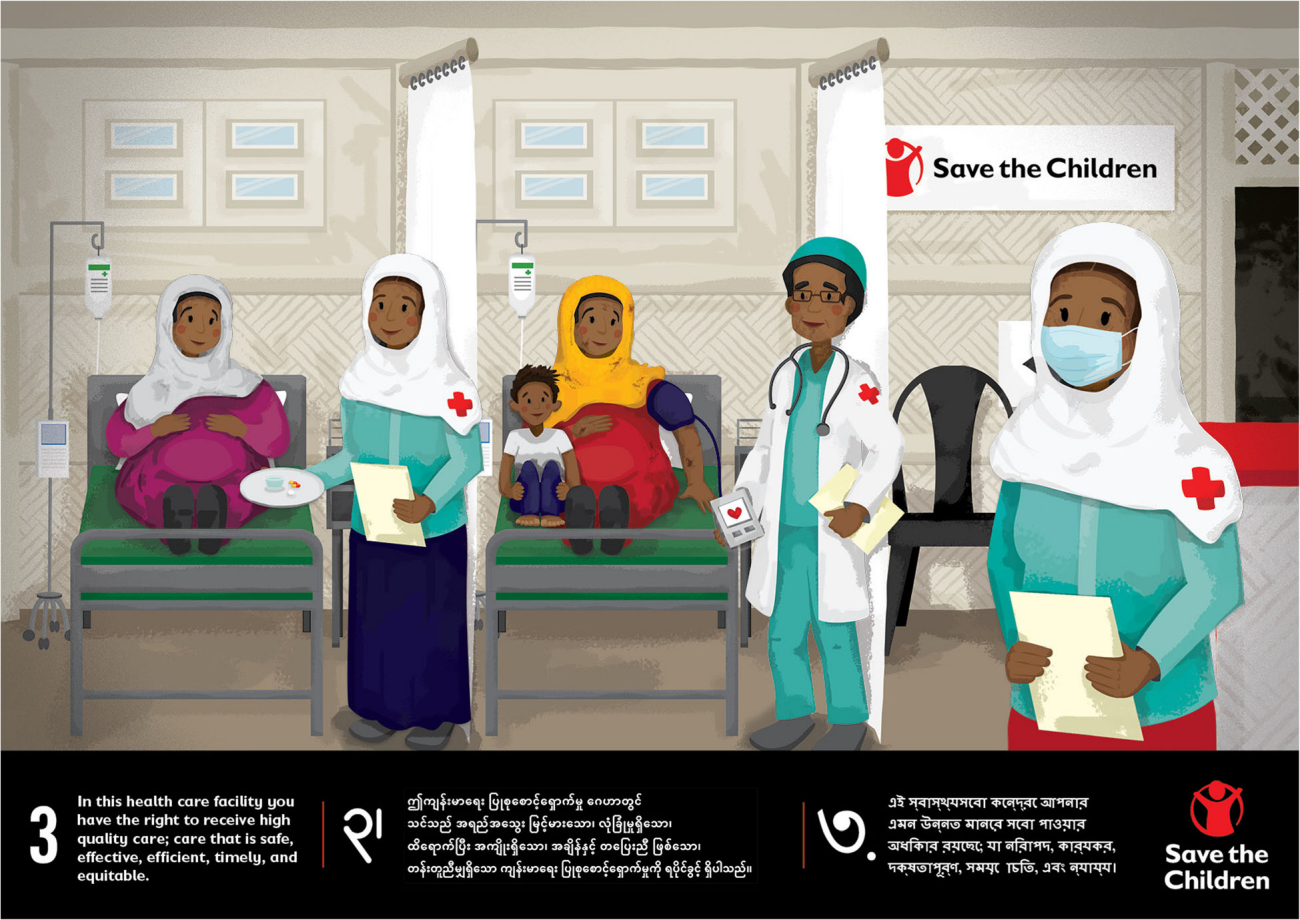
In this health care facility you have the right to receive high quality care

**Fig. 4:**
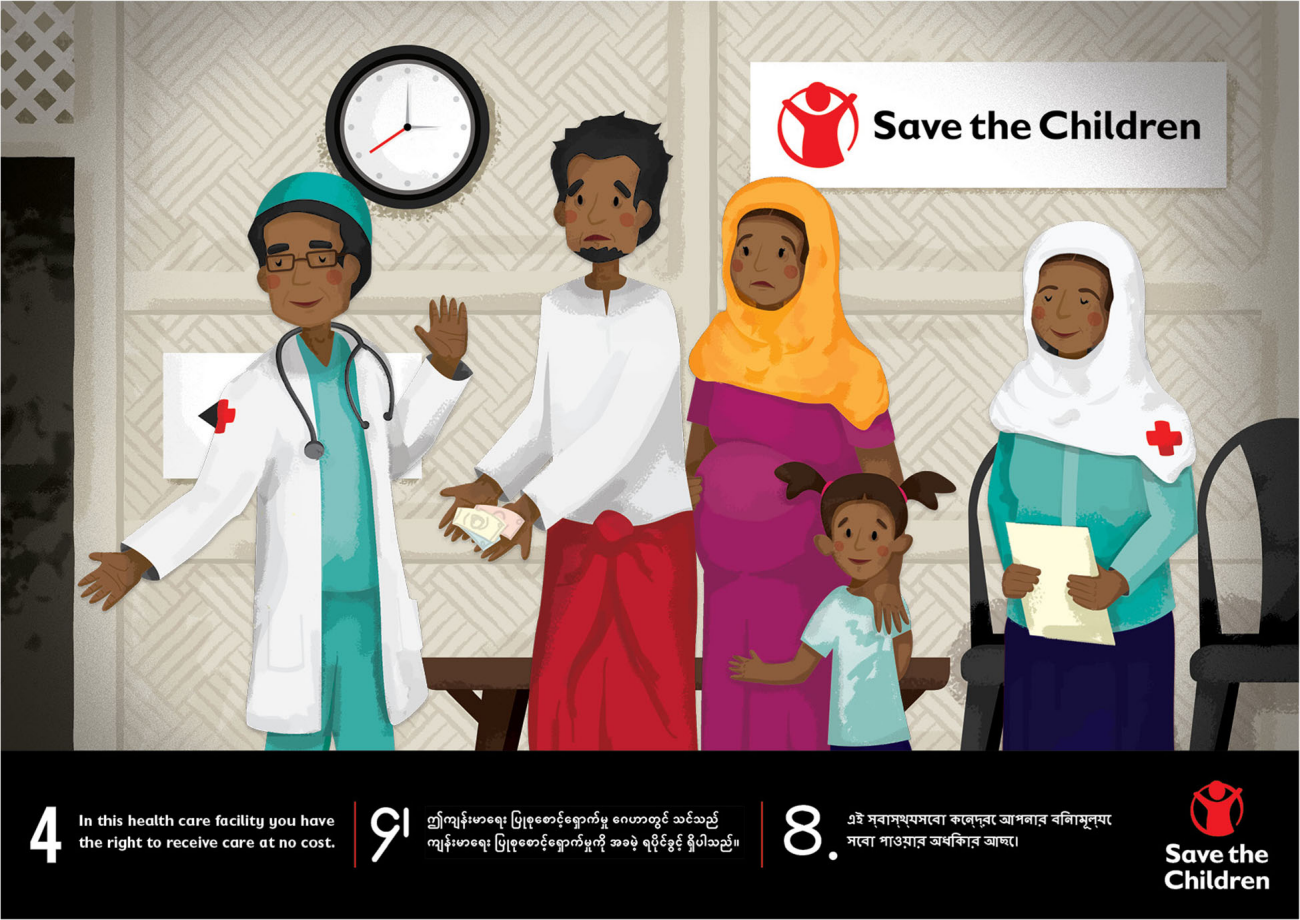
In this health care facility you have the right not to receive care at no cost

**Fig. 5:**
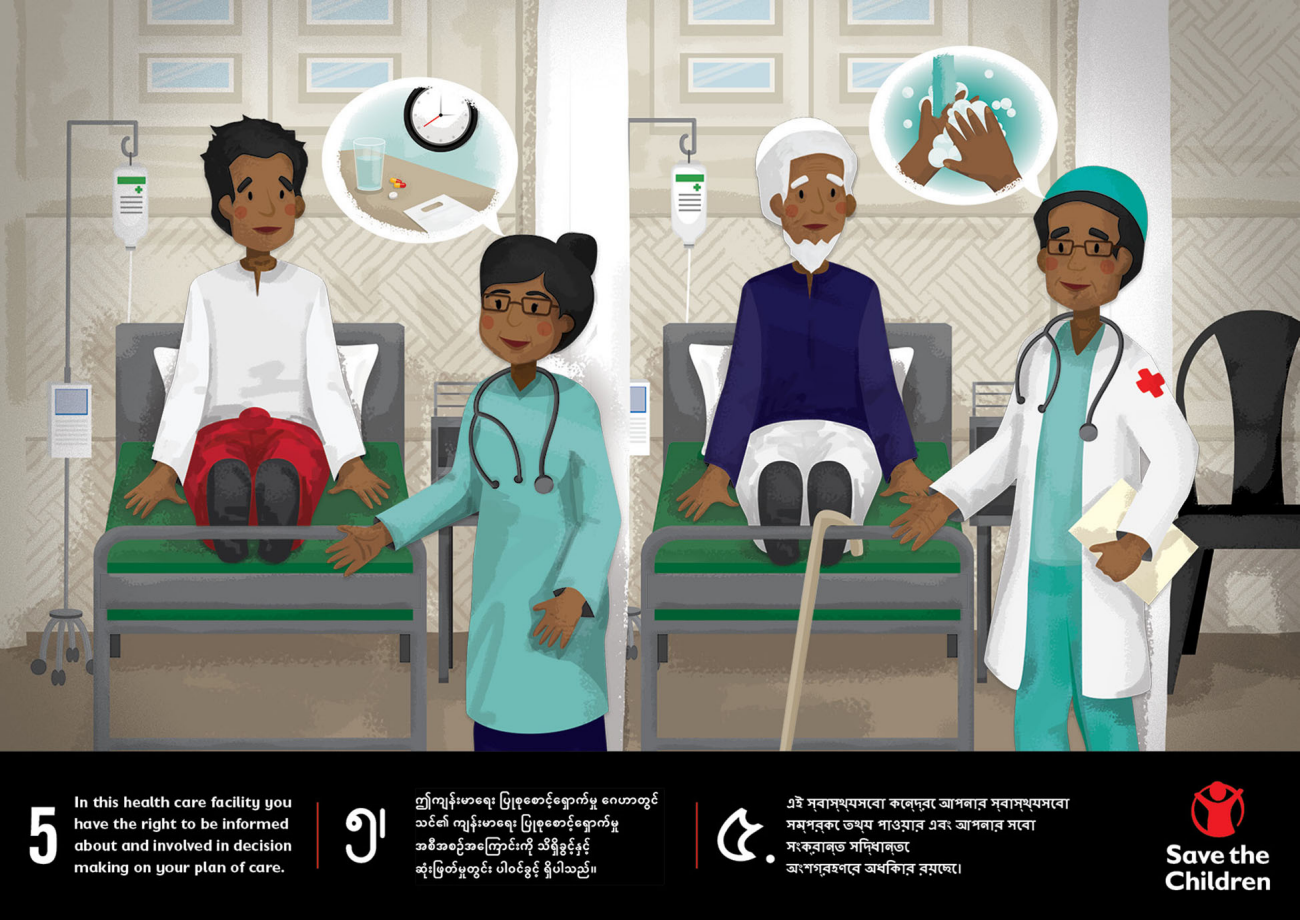
In this health care facility you have the right to be informed about and involved in decision-making on your plan of care

**Fig. 6:**
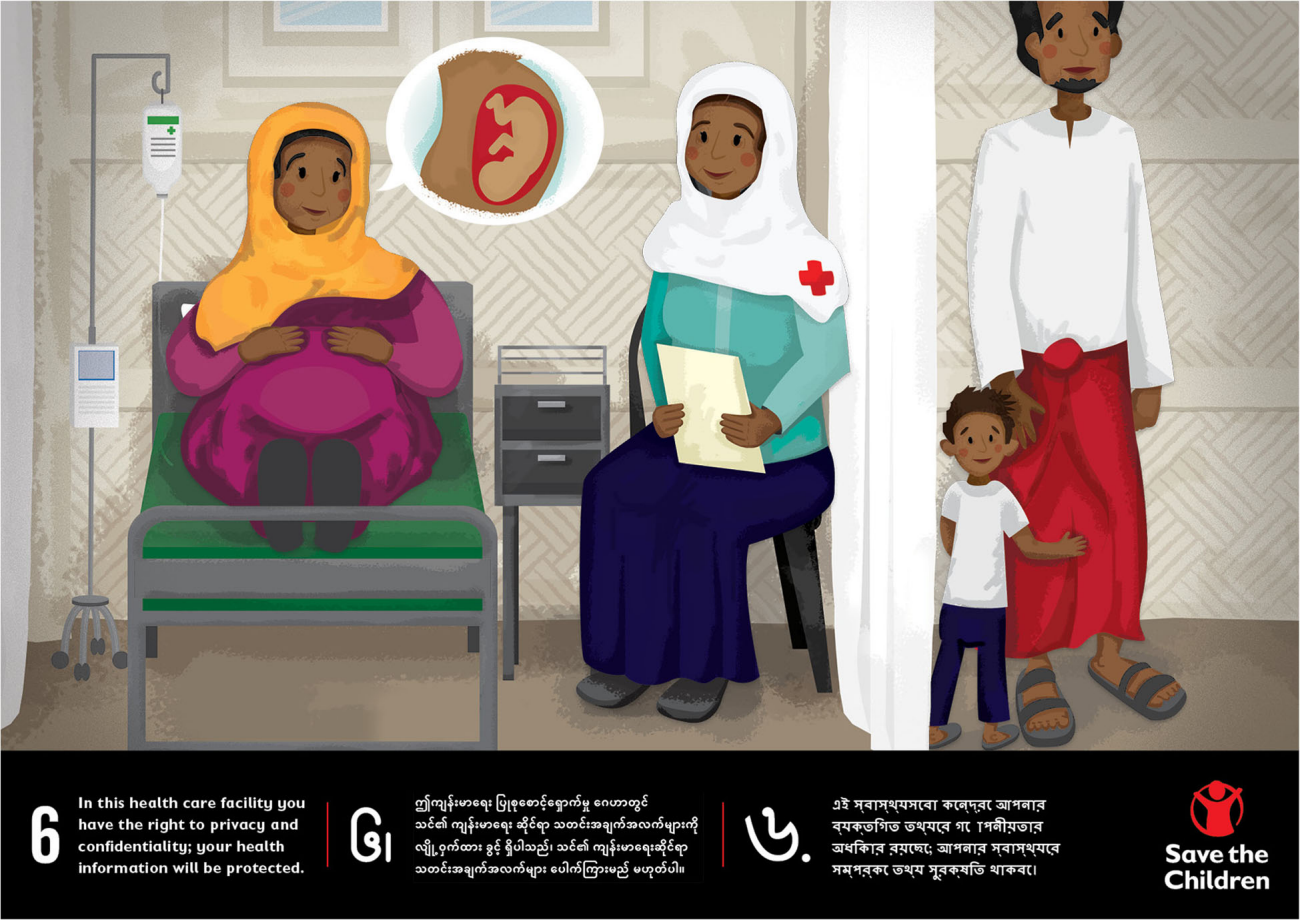
In this health care facility you have the right to privacy

**Fig. 7:**
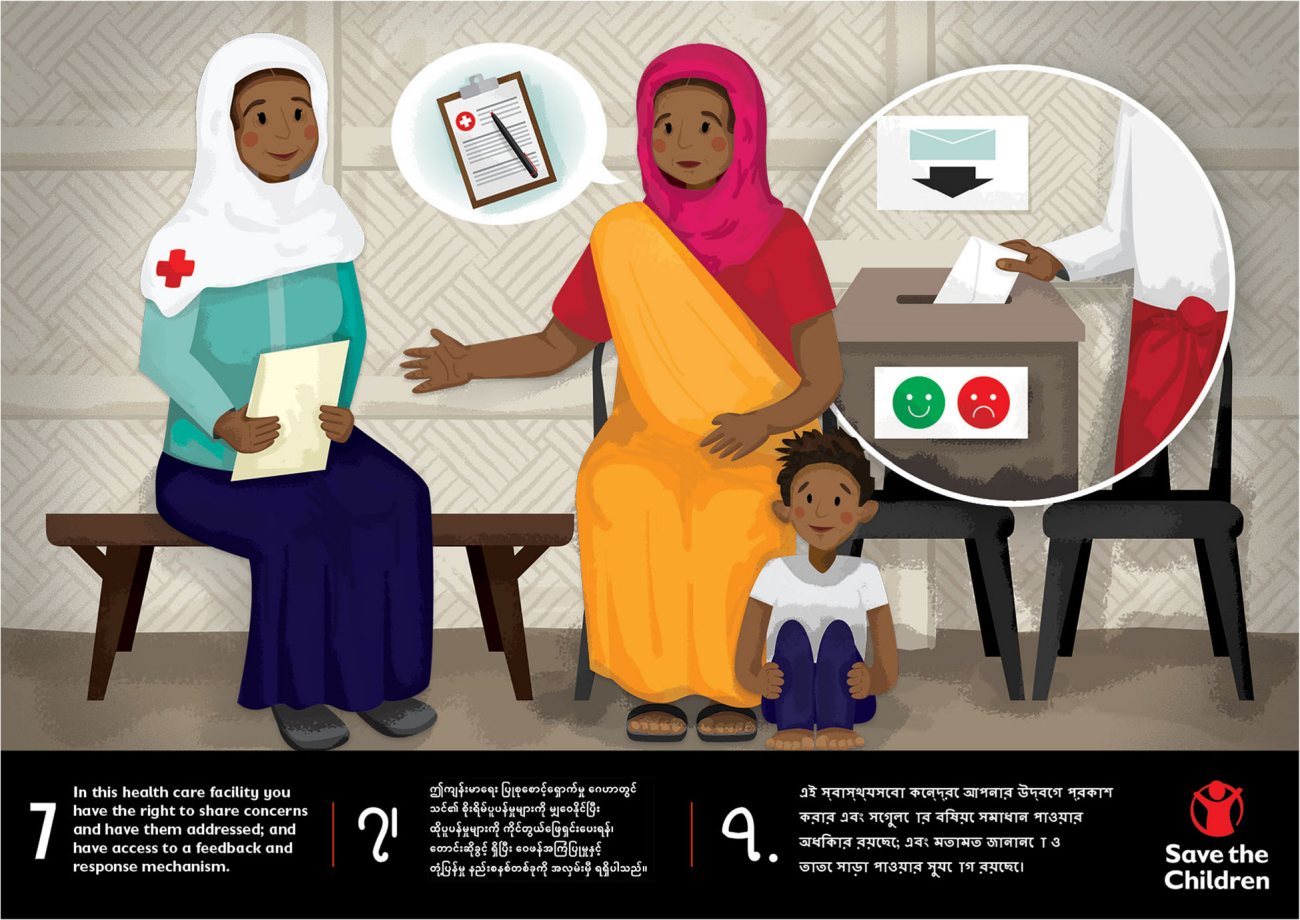
In this health care facility you have the right to share concerns and have them addressed

As the Save the Children SARI ITC was the dedicated referral facility for both Rohingya and the nearby host community, the charter needed to be accessible to both communities. Of particular concern was the accessibility of the charter to the Rohingya. A 2018 survey carried out by Translators Without Borders (TWB) found that of the 407 Rohingya respondents interviewed 66% indicated that they were unable to read or write in any language; this finding was largely confirmed through comprehension testing [6]. As neither Rohingya nor Chittagonian (a dialect of Bangla that is linguistically similar to Rohingya) have a standardised script, Rohingya who were able to read indicated a preference for written Burmese (32%), English (14%), and Bangla (11%) [6]. Thus, the Translators Without Borders study team recommended the use of these languages in all written communication.

In addition, the TWB report indicated that after spoken Rohingya, pictorial messages were the most widely understood medium and recommended that humanitarian communication efforts incorporate visual elements (such as illustration, animation, and video), created with the community to ensure they are appropriate and understood ([6] , p. 5). The report concluded that, “…language barriers and low access to media leave many Rohingya refugees without the critical and life-saving information they need to claim their rights, get the support they need, and make informed choices for themselves and their families” ([6] , p. 4).

To address these issues – particularly in the context of a novel disease outbreak that was eroding trust between the Rohingya and health actors [7, 8] – we sought to use graphic elicitation techniques to illustrate each of the seven rights contained within the charter through engagement with both Rohingya and host communities (Figs. 1, 2, 3, 4, 5, 6 and 7). Graphic elicitation has long been used in healthcare settings in developed contexts as a means to understand the health and wellness experiences of children (e.g. in children with cancer [9, 10], and children in chronic care [11]) and adults [12]. More recently the method has been described as a component of qualitative research interviewing, designed to stimulate dialogue [13–15]. Graphic elicitation has also been used to illustrate complex and largely abstract moral dilemmas faced by Kenyan fieldworkers [16, 17]. We drew heavily on this work to similarly depict the largely abstract concepts included in our patient charter.

## Methods

We designed our method to be carried out within the context of the COVID-19 response in Bangladesh and in observance of the requisite infection, prevention, and control (IPC) policies and procedures which limited the amount of interaction we were able to have with communities. The approach taken was based on and adapted from the graphic elucidation technique devised by Kingori in her work among data collectors in Kenya [16]. In our work, this method involved five discreet steps: 1) focus groups, 2) graphic elicitation, 3) graphic design, 4) feedback and revision, and 5) back-translation.

### Focus groups

It was agreed amongst the team that a focus group method was the most feasible given the constraints imposed both by IPC measures and the need to expedite the work. It was also felt that focus groups were the most appropriate means of eliciting feedback from a range of participants. Focus group members were selected from community contacts and purposively sampled for variation in gender and age. We carried out one focus group with the Rohingya (involving five women and five men) and one with the host community (involving eight women and six men). The larger venue in the host community allowed us to include more participants. All participants wore masks or other face coverings. We did not have the time or resources to carry out a focus group with children or adolescents.

As we required access to community members to help with the design of the posters we worked with the Humanitarian Coordination and Preparedness Team and the Communications and Media Team as both were experienced at coordinating and facilitating focus groups with the Rohingya and host communities. The facilitator was a native of Chittagong (Bangladesh) and spoke the local dialect. Though Chittagong is not always mutually comprehensible with Rohingya we chose to have her facilitate the focus groups as her experience communicating with both communities was extensive and other members of the team would be present to support in case there were any problems with translation.

To ensure a shared understanding of the charter within the team, a member of our MEAL team translated the charter into Bangla. The team reviewed and discussed the charter prior to undertaking the focus groups. During the focus groups the facilitator read each right in Bangla, and then discussed the concepts (e.g. dignity, equality, confidentiality) with the group taking special care to elicit discussion with those who were not as willing to speak in front of the group. Two other members of the team took notes during the discussion.

### Graphic elicitation

The facilitator asked participants how best to graphically represent each right, including the appearance of health staff and patients, their relative position, and their posture and demeanour (including appropriate gestures, etc). Participants were also asked to describe ways to depict inclusiveness (e.g. how best to illustrate disability, Rohingya and host community members, and people of differing socio-economic status). Following each focus group, the team discussed the dynamic withing the groups to determine if feedback from participants was equal and which elements of the charter were difficult for participants to either understand or describe.

### Graphic design

Notes from both focus groups were compiled and sent to a graphic designer who had prior experience creating illustrations based on feedback from community groups. The notes were also shared with the EHU Media and Communications Specialist who sent media content from Cox’s Bazar to the graphic designer to aid in the design of clothing and other context-specific visual elements. We agreed that the illustrations should not include negative messaging (e.g. a circle with a cross through it to indicate that something is prohibited) as this is discouraged in Save the Children’s global visual identity guidelines, and in much of the health promotion literature [18]. A member of the project team indicated that Rohingya women were not always aware that health facilities in the camps provide sexual and reproductive health services; thus, in the interest of depicting the provision of these services, we requested that the designer include pregnant women in the illustrations. The designer created a sketch of each right separately, using Adobe Illustrator and Adobe Photoshop (Adobe. 2019. Illustrator CC, Photoshop CC. Mountain View, CA: Adobe Inc). The final artwork was produced in Adobe InDesign (Adobe. 2020. InDesign CC: Release 15.0. Mountain View, CA: Adobe Inc). The final Bangla and Burmese translations were carried out by TWB who provided written translations and font files which were incorporated into each design alongside the English text.

The designer was careful to ensure that facial expressions helped to convey the message in each illustration. However, given the context, we opted to include face masks so that patients could see the continuity between what was illustrated and their real-world experiences. Thus, face masks were included in the illustration of health care workers where appropriate.

### Feedback and revision

We distributed the sketches amongst members of the MEAL team (including national and international staff), members of the EHU, and those involved in organising and facilitating the focus groups to request feedback. Feedback was sent to the designer who further modified the sketches. Colour was added to the sketches, and the posters were prepared for typesetting and branding. Our original intention had been to convene the original focus group members to review the sketches and provide feedback; however, a key member of the project team became ill and was unable to attend. To minimise delays, we decided to proceed without this step and the remaining team members reviewed the sketches and provided feedback.

### Back-translation

We had also intended to present the illustrations to community members who had not been part of the original focus group discussions to produce a back-translation (i.e. eliciting a description of the illustrations to determine the degree of agreement with the rights they are intended to depict). We felt this was an important component of the method owing to the abstract nature of the rights represented in the illustrations); however, as a result of a combination of factors (e.g. Eid al-Adha holidays, and the end of deployment for the first wave of EHU staff) we were unable to complete the back-translation of the illustrations as planned. 

## Results

Our method produced seven illustrations, each depicting one of the rights in the patient charter. Despite our concerns about the feasibility of illustrating largely abstract rights, participants in both groups were able to describe their rights visually. Our reflections on our method relate to: 1) our technique for eliciting feedback on the charter verbatim versus a broader discussion of concepts referenced within each right, 2) our decision to include both men and women in the same focus group, 3) our decision to ask focus group participants to describe specific features of each illustration and how this benefited the inclusivity of our illustrations, and 4) the potential of the focus groups to act as a means to introduce the charter to communities. The focus group discussions did not reveal any rights that were not included in our charter.

### Eliciting feedback

Both focus groups elicited rich discussion about the contents of the charter. However, the most evocative content was garnered, not from discussion of the rights themselves, but from structured discussions of participants’ experience and understanding of some of the concepts contained in the charter. Examples of how ‘dignity’ and ‘equality’ manifest, or not, in a health care setting were particularly useful. For example, discussion of experiences in healthcare settings in which dignity was compromised provided a helpful starting point for describing how to illustrate dignified care. On reflection, discussing rights more generally, and asking participants to provide examples from their own experiences, may have been a better starting point than reading out each right.

### Focus group composition

Owing to limited time and a narrow window of opportunity we could only carry out two focus groups. As the Rohingya and host community were accessible at different locations we opted to carry out one focus group with the Rohingya, and one with the host community. This meant that both groups included men and women. The facilitator was able to elicit feedback, through regular prompting, from both men and women (who were less forthcoming than the men in the Rohingya group, and who were being actively discouraged from contributing by the men in the host community group); however, a less experienced facilitator may have struggled to engage the women equally in the discussion.

### A focus on details

Differences in clothing between the Rohingya and host community were emphasised during both focus groups as participants were eager to ensure that their respective communities were represented in the illustrations. Participants were also forthcoming about how health care workers should be dressed, where they should be standing, and various nuances relating to comportment (e.g. Rohingya infrequently ‘point’, rather they draw attention to people or objects using an open hand). Though communicating this information took time, and resulted in lengthy fieldnotes, this information was critical in ensuring that the illustrations were culturally appropriate. The issue of stature was discussed repeatedly, and the Rohingya in particular requested that Rohingya people depicted in the illustrations not be positioned ‘lower’ than health care workers. Thus, we were mindful of relative height in our illustrations. Though this information did not always appear to relate directly to the rights we were attempting to depict, it was important to participants in both communities and, in retrospect, was critical in ensuring the localisation of the rights included in the charter.

### The value in the process

Participants in the host community expressed surprise that they had rights as patients in a health care setting. The focus groups were, inadvertently, a good opportunity to discuss these rights and to answer any questions participants had about how we developed the charter, to whom the rights applied, etc. The focus groups themselves had value, not only in terms of enabling graphic elicitation, but also in allowing us to introduce the patient charter to community groups. In a context in which much information is spread via word of mouth the process maybe an effective means to increase the awareness of, and dialogue around, patient rights. 

### Limitations

Our method was difficult to execute as we intended. Despite our desire to establish how accurately the sketches represented what participants were trying to convey, we were unable to reconvene the original focus groups, nor were we able to carry out the back translation of the images (to determine if community members could tell us what was being depicted in the illustrations without referring to the rights as written). Operationalising even the most basic activities in an emergency is logistically complicated; however, in this setting routine operational challenges were compounded by the nature of the emergency (and the associated infection prevention and control measures) and the standard restrictions on camp access (e.g. access hours were limited for international staff). These factors, in addition to competing demands and time constraints, prevented us from determining if the illustrataions faithfully depicted the visual elements described to us, and from carrying out the back-translation as originally planned.

Despite early outbreak modelling predictions there have been few laboratory confirmed cases of COVID-19 within the catchment of our SARI ITC, and amongst the Rohingya population more broadly [8]. We have thus been unable to measure the impact of our patient charter (e.g. in terms of its effect on access to services, patient satisfaction, and/or patient safety; or on patients’ ability to recall the components of the charter or their views of adherence to charter by medical personnel) and the accompanying illustrations amongst our patients. Ideally, we would have been able to establish the acceptability and accessibility of the materials (e.g. though patient exit interviews), and to make further changes to the materials based on patient feedback.

Finally, we were unable to identify people living with disabilities for inclusion in our focus groups, nor did we include children or adolescents.

## Discussion

Despite the difficulties implementing our method as planned we were able to work with both Rohingya and host community members to provide culturally appropriate and contextualised illustrations to accompany the rights in our patient charter. We believe this would be an important exercise even amongst populations with higher literacy, as the illustrations also serve both to communicate how rights are understood by affected populations, and to establish normative expectations of their interaction with health services. This is particularly important for health services provided by emergency medical teams which may not have experience operating in the contexts to which they have deployed, as well as for teams that draw on roster staff who may deploy infrequently.

Though we tried to make the rights in the charter accessible through discussion with the community and by creating accompanying illustrations, we did not engage with the community on the rights themselves. We set out to develop a standard patient charter that would represent a commitment by the EHU to respecting and providing for patient rights; however, the alternative would possibly be to develop a charter for each emergency response in consultation with affected communities in order to ensure that the rights themselves are contextually appropriate. It was clear from the focus groups that some rights were taken for granted (e.g. the right to receive healthcare at no cost) but others elicited more nuanced discussion (e.g. the issue of dignity) which could have provided the foundation for a more deliberative articulation of rights. However, as we found it difficult to carry out our planned method in an emergency setting (albeit one complicated by stringent IPC procedures) it may be too ambitious to co-produce the charter itself without additional dedicated resources.

Given our realisation that the focus groups were an opportunity to introduce the patient charter to participants we believe we missed an opportunity to involve community leaders or other influential community members in the focus groups. Additionally, we could have provided a copy of the patient charter in English, Bangla, and Burmese for participants to keep and to discuss with other community members. Translators Without Borders has suggested that humanitarian agencies develop illustrated brochures and leaflets that can be shared so that those with lower levels of literacy can ask trusted friends or family members to help them understand the information; “[g]iven both access and privacy concerns, women in particular may benefit from this approach, which can complement mass communication materials such as posters” ([6] , p. 30).

Additionally, though our discussion of patient rights was fruitful and participants were engaged and content to discuss their experiences, it would have been useful to have a mental health and psycho-social support (MHPSS) adviser present in case any of the concepts being discussed triggered distressing memories (e.g. in the context of a discussion about experiences of discrimination).

Finally, given how quickly context changes in emergency settings we endorse the regular review of the images as they may lose relevance over time.

## Conclusion

Informing and educating patients, the public, and health care workers about patient rights should be a dynamic and iterative process to ensure a contextually appropriate charter of patient rights. Agreeing that a patient charter was a key accountability tool – one that was likely to be largely inaccessible to many affected people in Cox’s Bazar, Bangladesh – we undertook focus groups with both Rohingya and host community members. The focus groups produced rich feedback from which we were able to create illustrations to depict the rights contained in the charter. Despite our best efforts we were unable to execute our method as planned; however, our experience has highlighted some of the challenges in carrying out graphic elicitation (and potentially other deliberative methods) in the context of a humanitarian crisis and some of the opportunities to engage affected populations whilst observing infection and control procedures.

## Data Availability

Please note that the posters are subject to copyright belonging to Save the Children. Unbranded versions of the posters in Adobe InDesign format are available on request. Permission for use and/or any distribution right of the posters including any modification of the posters is subject to obtaining prior written approval, granted at the sole discretion of Save the Children.

## Abbreviations

AAR: After action review
CIC: Camp in charge
EHU: Emergency health unit
FDMN: Forcibly displaced Myanmar nationals
ITC: Isolation and treatment centre
MEAL: Monitoring, evaluation, accountability and learning
NHS: National health service
SARI ITC: Severe acute respiratory infection
TWB: Translators without borders
